# Advances in Magnetic Sensors and Their Applications

**DOI:** 10.3390/s25175590

**Published:** 2025-09-08

**Authors:** Nicholas Sammut, Marco Calvi

**Affiliations:** 1 Department of Microelectronics and Nanoelectronics, Faculty of ICT, University of Malta, MSD 2080 Msida, Malta; 2 Photon Science Division, Paul Scherrer Institute, Forschungsstrasse 111, 5232 Villigen, Switzerland; marco.calvi@psi.ch

Magnetic sensors are fundamental to a vast array of scientific and technological endeavours, permeating disciplines from fundamental physics [1,2] and medical diagnostics [3,4,5] to industrial automation [6,7], automotive engineering [8,9] and space exploration [10,11]. These devices, capable of detecting and measuring magnetic fields, play a crucial role in enabling a multitude of modern technologies that rely on precise magnetic information. The relentless pursuit of higher sensitivity, enhanced accuracy, greater miniaturization, and broader versatility continues to drive significant innovation in this critical field, pushing the boundaries of what is possible in various applications. This Special Issue provides a comprehensive forum for presenting cutting-edge research, development, and ground-breaking innovations pertaining to magnetic sensors and their diverse magnetic sensing applications.

Our aim was to curate a collection of articles that collectively highlight the state of the art and future directions in magnetic sensing, directly reflecting the innovative work presented in this Special Issue. The contributions cover a wide spectrum of topics, including the following:

- Novel magnetic sensor designs and materials, such as those employing amorphous microwire cores or advanced Hall element arrays for high-resolution mapping. These innovations contribute to the development of more sensitive and reliable magnetic field detection.
- Advanced sensing systems for specific industrial and engineering applications, including sophisticated tactile sensing systems utilizing magnetorheological structures and robust devices for wire rope breakage detection in high-speed operations. The issue also features position detection systems crucial for moving magnet linear motors.
- Magnetic sensors in various medical applications, showcasing their transformative potential in healthcare. This includes magnetostatic simulation and the design of novel radiofrequency coils for magnetic resonance imaging (MRI), in silico studies on frequency mixing magnetic detection (FMMD) for biosensing, and the design of wireless implantable sensors for abdominal aortic aneurysm monitoring. Furthermore, it covers phantom-based approaches for comparing magnetoencephalography (MEG) systems and line-source simulations for microwave imaging in breast lesion detection.
- Characterization and measurement techniques for magnetic components and fields, such as online methods for excitation impedance of current transformers and theoretical revisits of electromagnetic wave scattering by metal isotropic bodies with magnetic sensor excitation.
- Innovative approaches to wireless sensing and energy harvesting for magnetic field measurement, including RFID sensors with integrated energy harvesting for DC magnetic fields.
- Development of high-sensitivity magnetic sensors, exemplified by superconducting quantum magnetometers based on the flux focusing effect, which are critical for detecting extremely weak magnetic signals.
- Modelling and simulation techniques for magnetic sensing, underpinning the design and optimization of these advanced systems.

This Special Issue proudly features fourteen significant contributions that collectively demonstrate the extensive and innovative research being conducted in the field of magnetic sensing. In Contribution 1, Yu-Jin Park, Bo-Gyu Kim, Eun-Sang Lee, and Seung-Bok Choi present “A Novel Tactile Sensing System Utilizing Magnetorheological Structures for Dynamic Contraction and Relaxation Motions”. This work introduces a soft tactile sensor utilizing a magnetorheological elastomer (MRE) with embedded permanent magnets. The sensor leverages the dynamic deformation and magnetic force of the MRE under an applied magnetic field to provide haptic feedback, demonstrating its potential for creating more intuitive human–machine interfaces.

In Contribution 2, Zhou Zhou, Xiuheng Zhang, Ran Deng, Lu Han, Meng Zhou, Zhuangzhuang Ma, Xiangdong Chang, and Yuxing Peng investigate “Research on a Wire Rope Breakage Detection Device for High-Speed Operation Based on the Multistage Excitation Principle”. Their research focuses on developing a robust non-destructive testing method for wire ropes, critical for safety in high-speed industrial applications. The proposed device uses a multistage excitation magnetic circuit and a Hall sensor array to improve defect detection accuracy and reduce false alarms, even at high operational speeds.

In Contribution 3, Giulio Giovannetti, Marcello Alecci, and Angelo Galante contribute “Magnetostatic Simulation and Design of Novel Radiofrequency Coils Based on Transverse Field Current Elements for Magnetic Resonance Applications”. This paper explores the design of new RF coils for MRI, aiming to achieve more uniform magnetic fields and improved signal-to-noise ratios. They employ magnetostatic simulations and a transverse field current element approach to optimize coil geometries for various magnetic resonance applications, including those involving inhomogeneous fields.

In Contribution 4, Bin Wang, Weizhi Xu, Xiaoping Zheng, Sida Jiang, Zhong Yi, Peng Wang, and Xiaojin Tang analyze the “Performance of Fluxgate Magnetometer with Cu-Doped CoFeSiB Amorphous Microwire Core”. This study focuses on enhancing the sensitivity and reducing the noise of fluxgate magnetometers by incorporating a novel Cu-doped CoFeSiB amorphous microwire as the core material. Their findings demonstrate improved magnetic properties, making these sensors suitable for high-precision magnetic field measurements in challenging environments.

In Contribution 5, Ulrich M. Engelmann, Beril Simsek, Ahmed Shalaby, and Hans-Joachim Krause delve into “Key Contributors to Signal Generation in Frequency Mixing Magnetic Detection (FMMD): An In Silico Study”. This in-depth computational study identifies the primary physical parameters influencing signal generation in FMMD, a technique used for highly sensitive biosensing with magnetic nanoparticles. Understanding these factors is crucial for optimizing FMMD systems for various biomedical diagnostic applications.

In Contribution 6, Mengying Gan, Hongsen You, and Jiansheng Yuan present an “Online Measurement Method and System of Excitation Impedance of Current Transformers Based on Norton’s Theorem and Differential Method to Measure Difference of Two Currents”. This paper addresses the critical need for accurate online monitoring of current transformer performance in power systems. Their proposed method, based on Norton’s theorem, allows for the non-invasive and precise measurement of excitation impedance, which is vital for ensuring grid stability and efficiency.

In Contribution 7, Nuno P. Silva, Adnan Elahi, Eoghan Dunne, Martin O’Halloran, and Bilal Amin detail the “Design and Characterisation of a Read-Out System for Wireless Monitoring of a Novel Implantable Sensor for Abdominal Aortic Aneurysm Monitoring”. This work focuses on developing a wireless, battery-free read-out system for an implantable pressure sensor, enabling continuous and long-term monitoring of abdominal aortic aneurysms. The system employs magnetic resonant wireless power transfer and demonstrates robust performance in preclinical setups, offering a less invasive follow-up solution for patients.

In Contribution 8, Tan Zhou, Jiangwei Cai, and Xin Zhu introduce “An Advanced Hall Element Array-Based Device for High-Resolution Magnetic Field Mapping”. This research presents a sophisticated Hall sensor array designed for the precise and high-resolution mapping of magnetic fields. The device integrates multiple Hall elements to capture detailed magnetic field distributions, which has wide applications in quality control, material characterization, and non-destructive testing.

In Contribution 9, Panayiotis Vafeas provides “A Revisit of Electromagnetic Wave Scattering by a Metal Isotropic Body in a Lossless Environment with Magnetic Sensor Excitation”. This theoretical paper re-examines the fundamental principles of electromagnetic wave scattering from metallic objects when excited by magnetic sensors. It offers analytical and numerical solutions for understanding the scattered fields, which is essential for designing and interpreting data from various magnetic sensing and imaging systems.

In Contribution 10, Antonio Vettoliere and Carmine Granata describe a “Superconducting Quantum Magnetometer Based on Flux Focusing Effect for High-Sensitivity Applications”. This paper details the development of a highly sensitive DC superconducting quantum interference device (SQUID) magnetometer that utilizes a large washer-shaped superconducting loop to focus magnetic flux. This design achieves excellent magnetic field noise performance, making it suitable for demanding applications like magnetoencephalography (MEG) and other low-field measurements.

In Contribution 11, Jun Wang, Xiang Chen, Quyan Chen, Qing Xi, and Haiyang Sun explore a “Position Detection System for Moving-Magnet Linear Motors Based on a Magnetoresistive Sensor Array”. This paper proposes a high-precision position detection system crucial for the accurate control of moving magnet linear motors. By employing an array of magnetoresistive sensors and a specialized algorithm, the system effectively compensates for magnetic field non-uniformities, achieving high accuracy in real-time position tracking.

In Contribution 12, Daisuke Oyama and Hadi Zaatiti present a “Phantom-Based Approach for Comparing Conventional and Optically Pumped Magnetometer Magnetoencephalography Systems”. This study introduces a standardized phantom-based methodology for directly comparing the performance of conventional SQUID-based MEG systems with newer optically pumped magnetometer (OPM) MEG systems. Their approach provides a controlled environment to assess sensitivity, spatial resolution, and overall measurement fidelity, contributing to the validation and adoption of OPM technology in neuroimaging.

In Contribution 13, Shijie Fu, Greg E. Bridges, and Behzad Kordi develop an “RFID Sensor with Integrated Energy Harvesting for Wireless Measurement of dc Magnetic Fields”. This innovative work describes a battery-free RFID sensor capable of wirelessly measuring DC magnetic fields by integrating an energy harvesting module. The sensor is designed for applications such as monitoring high-voltage direct-current (HVdc) transmission lines, offering a low-cost and maintenance-free solution for remote sensing.

In Contribution 14, Navid Ghavami, Sandra Dudley, Mohammad Ghavami, and Gianluigi Tiberi introduce “A Line-Source Approach for Simulating MammoWave Microwave Imaging Apparatus for Breast Lesion Detection”. This paper proposes an analytical simulation method for the MammoWave apparatus, a microwave imaging system used for breast cancer detection. The line-source model offers a computationally efficient alternative to full-wave simulations or phantom measurements, providing a valuable tool for optimizing the system’s design and understanding its detection capabilities for breast lesions.

These contributions underscore the rapid advancements and the interdisciplinary nature of research in magnetic sensors and their pervasive magnetic sensing applications. They not only address current technological challenges but also pave the way for novel discoveries and foster scientific collaborations across various domains.

We extend our deepest gratitude to all the authors for their high-quality contributions, which reflect their dedication and expertise in the field. Our sincere thanks also go to the diligent reviewers, whose invaluable time and rigorous efforts were instrumental in maintaining the high scientific standards and integrity of this Special Issue. Finally, we appreciate the excellent support from the editorial team at *Sensors* for their commitment throughout the publication process.

We trust that this Special Issue will serve as a valuable resource and an inspiring read for researchers, engineers, and practitioners engaged in the exciting field of advanced magnetic sensors and their transformative applications.
